# LncRNAs as Potential Therapeutic Targets in Thyroid Cancer

**DOI:** 10.31557/APJCP.2020.21.2.281

**Published:** 2020

**Authors:** Zeeshan Javed, Faiez Ahmed Shah, Sadegh Rajabi, Qamar Raza, Zaheer Iqbal, Mukhtar Ullah, Touqeer Ahmad, Bahare Salehi, Mehdi Sharifi-Rad, Raffaele Pezzani, Farooq Yaqoob, Haleema Sadia, Marcello Iriti, Javad Sharifi-Rad, William C Cho

**Affiliations:** 1 *Office for Research Innovation and Commercialization Lahore Garrison University, *; 4 *Institute of Biochemistry and Biotechnology,University of Veterinary and Animal Sciences, *; 5 *Center for Excellence in Molecular Biology, University of the Punjab Lahore, Lahore, *; 2 *Department of Biotechnology, Quaid-i-Azam University, Islamabad, Pakistan, *; 3 *Department of Clinical Biochemistry, School of Medicine, *; 1 *Phytochemistry Research Center, Shahid Beheshti University of Medical Sciences, Tehran,*; 6 *Student Research Committee, School of Medicine, Bam University of Medical Sciences, Bam, *; 7 *Department of Medical Parasitology, Kerman University of Medical Sciences, Kerman, Iran, *; 9 *8OU Endocrinology, Dept. Medicine (DIMED), University of Padova, via Ospedale 105, 35128, *; 7 *AIROB, Associazione Italiana per la Ricerca Oncologica di Base Padova, *; 10 *Department of Agricultural and Environmental Sciences, Milan State University, 20133 Milan, Italy, *; 12 *Department of Clinical Oncology, Queen Elizabeth Hospital, 30 Gascoigne Road, Hong Kong, China. *

**Keywords:** LncRNA, treatment, genetic alterations, thyroid cancer

## Abstract

Thyroid cancer (TC) is the most common cancer of endocrine system. TC can be subdivided into 4 different entities, papillary, follicular, medullary and anaplastic thyroid cancer. Among them, anaplastic thyroid cancer has the poorest prognosis. Exploring new therapeutic approach may entail favorable prediction as well as increasing overall survival rate of patients. Long non-coding RNAs (lncRNAs), have vast implications in different cancer types. Although they are not transcribed into proteins, they can act as a harness in regulating a plethora of biological functions. They have been implicated in a decisive role in gene expression via modulation of both coding and non-coding RNAs. This article discuss the multi-facet role of lncRNA in thyroid cancer biology.

## Introduction

Among malignant tumors, thyroid cancer (TC) has been the most common cancer of endocrine gland. It has been estimated that the overall incidence of the TC in the US alone has escalated from 2.4% per year to 6.6% per year (Solomon et al., 2019). The incidence of TC has been escalating for the past thirty years. In the US alone endocrine cancers prevalence is mounting with roughly 1-1.5% of all newly diagnosed cancers every year (Orosco et al., 2019). The annual percentage change of thyroid tumors is increasing in the globe except Africa at where detection is not popular (Kilfoy et al., 2009). Appertaining to these findings it has been perceived that TC is the fifth most prevalent type of anomaly in females and it severely affects women under the age 45 years (Guerra et al., 2014). Several factors have been investigated for this robust high variability of thyroid tumors. Genetic predispositions an environmental factors may account for the disease access to the medical facilities are the key for the diagnosis of TC. Among thyroid tumors, papillary thyroid cancer is more common in black females while male proportions were found to be highest in percentage in white males 6.3-7.1%. Similar percentage prevalence was observed in various male populations such as the Hispanic 6.7%, Asian 6.4% and 8.4% in African males (Pellegriti et al., 2013). TC account for 0.3% mortality incidence in the US and rates of mortalities are similar around the world. TC incidence is 10.5 per 100,000 in the US (Alok Pathak et al., 2013). Based on its cellular origin, TC is classified into 4 different entities, papillary (PTC), follicular (FTC), medullary (MTC) and anaplastic thyroid carcinoma (ATC) (Trimboli et al., 2006). Papillary carcinoma is the most common one, has an incidence rate ranging between 80-85%, with the highest prognostic rate in ten years with a survival ratio >90-95% with low metastatic potential. Follicular carcinoma is the second most prevalent tumor of thyroid followed by medullary and anaplastic cancers (Aboelnaga and Ahmed, 2015).

A surfeit of cellular as well as genetic processes such as genetic aberrations, epigenetics, and dysregulated cellular signaling have been reported to orchestrate the tumorigenesis of TC (Murugan et al., 2017). Indeed, different cellular pathways have been found altered in TC, such as the ERK/MAPK/PI3K, RAF/Ras, JAK/STAT and Wnt/beta-catenin (Liu et al., 2018b). These cellular signalings modulation enhances cellular proliferation and accelerates tumorigenesis. Nowadays, it has become easy to understand the pattern of DNA methylation in TC (Bhan et al., 2018) and histone modifications implicated in the spread of TC (Joo et al., 2018). However, the exact insight of how epigenetic framework reconstitutes the abrogated cell signaling in TC has only partially explored (Lamartina et al., 2018). Thus, it is necessary to find new diagnostic biomarkers to characterize TC. Similarly, the discovery of novel biomarkers as therapeutic targets is still an important issue in TC. Accordingly, the main purpose of this review is to summarize the different roles of lncRNA in TC biology. 

*LncRNAs and Thyroid Cancer*

**Figure 1:**
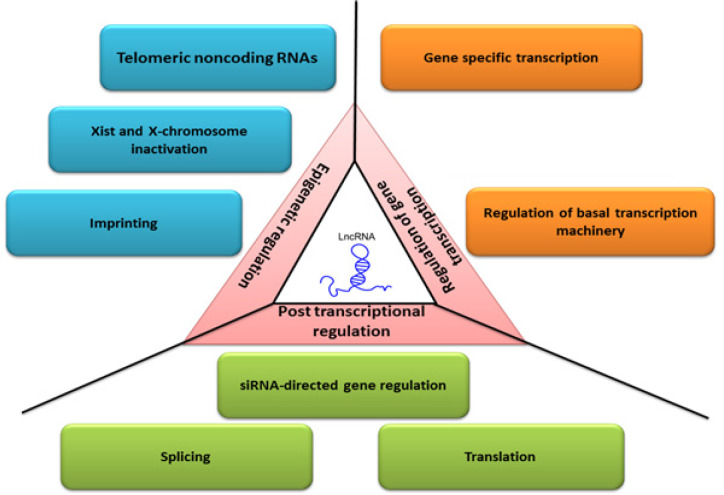
Functions and Mechanism of Action of lncRNAs in Different Biological Processes such as Transcription, Post-Transcription and Epigenetic Regulation

LncRNAs are at least > 200 bp in these tiny molecules were once referred to as “junk DNA.” LncRNAs encompasses 80% of the human genome that remains untranslated. They are distinctively different from the coding regions in their biogenesis and functions (Hangauer et al., 2013; Starren et al., 2013). They also entail unique properties from the coding RNAs which confer them incomparable structural and functional superiority over the coding genome. Despite their exceptional physiology, they share some similarities with other RNA molecules such as microRNAs, small nucleolar RNAs, etc. Yet, they have been shown to play a pivotal role in eukaryotic mammalian cells (Bierhoff et al., 2011). They are transcribed by RNA polymerase II and in some cases by RNA pol III. They have been reported as mediators and regulators of epigenetic activation of specific oncogenes (Guttman et al., 2009; Gutschner and Diederichs, 2012). Owing to these characteristics, lncRNAs are a hallmark in tumor biology of TC as they can promote cellular proliferation. The interplay between lncRNAs and oncogenes may lead to tumor progression (Zheng et al., 2016). Understanding this area is promising for impeding TC progress or fostering new strategies for TC treatment (Jalali et al., 2013; Liao et al., 2017). They have been linked to unabated cell growth, prolonged metastasis and upregulation of oncogenes, which are summarized in Figure 1. They act via either activation of promoters or suppressors of oncogenes. Consequently, they mediate a variety of biological cell processes. 

*Molecular Mechanism of LncRNAs in Thyroid Cancer*

Despite the advancements in the fields of bioinformatics, proteomics and genome-wide association analysis (GWAS), little is known on the exact mechanisms by which lncRNAs modulate cellular physiology of endocrine cells. However, genome-wide analysis profiles have revealed a deep insight into the molecular mechanisms of lncRNAs in TC. LncRNAs have been implicated in the development and differentiation of thyroid cells. Lan et al., (2015) using GWAS and qRT-PCR expression profiling characterized more than 10 different lncRNAs in 62 PTC patients. They revealed the modulatory effects of these lncRNAs on gene function using gene ontology and KEGG (Kyoto Encyclopedia of Genes and Genome) pathway analysis. Based on their algorithms, they concluded that lncRNAs modulated gene expression via cis or trans targeting consequently influencing key processes like growth and differentiation of PTC. Furthermore, their findings indicated that lncRNAs were the sole element in harnessing carcinogenesis in PTC. Microarray-based study has emphasized two lncRNAs deregulations, such as XLOC_051122 and XLOC_00604. Overexpression of these two lncRNAs promotes carcinogenesis via activation of BRAF and LNM genes in a synergistic manner, suggesting that these two lncRNAs could be a useful prognostic marker for PTC (Liyanarachchi et al., 2016). In the light of these findings, it can be concluded that there exists a vast range of lncRNAs that modulate the expression of numbers of genes involved in differentiation as well as apoptosis. Oncogenic lncRNAs have far more implications in determining the deteriorations of tumor cell infrastructure and thus could be utilized as a useful tool for diagnostic, prognostic and therapeutic targets. In TC majority of oncogenic lncRNAs exert their influence via targeting of E-cadherin. E-cadherin promoter EZH2 (polycomb enhancer of Zeste homolog 2) recruitment by either NEAT1 (Nuclear enriched abundant transcript 1), BANCR (BRAF-activated lncRNA) or PVT1 (plasmacytoma variant translocation 1), MALAT1 (Metastasis-associated lung adenocarcinoma transcript 1) and HOTAIR (HOX transcript antisense RNA) results in the activation of thyroid stimulating hormone receptor (TSHR) which in turn promotes the expression of Cyclin D1 that triggers cellular proliferation in TC (Zheng et al., 2016). 

*Involvement of Oncogenic lncRNAs in Thyroid Cancer*

LncRNAs do not code for proteins and the best way to understand their function is through expression analysis (Derrien et al., 2012). Tumor diagnosed at an early stage could provide extended survival or characterize pathological features of the tumor as well. LncRNAs have the potency to be detected in blood as well as in tissue well, thus making them a diagnostic tool that can be easily detected (Schwarzenbach et al., 2011). In addition, lncRNAs could also aid in the diagnostic assessment of PTC via ultrasonography. An extracellular protein highly expressed on cell surface known as fibronectin 1 promotes transcription of the NONHSAT076754 a long non-coding RNA. NONHSAT076754 has been involved in cellular processes such as cell adhesion, migration and injury response. Xiang et al., (2017) studied the involvement of NONHSAT076754 in PTC. Their findings revealed that NONHSAT076754 promoted migration and invasiveness in PTC and addition of NONHSAT076754 along with ultrasonography led to an increased in the diagnostic credibility of PTC. Therefore, NONHSAT076754 could be used as a valuable auxiliary diagnostic biomarker with ultrasonography for the prediction of cervical lymph node metastasis in PTC. All these evidence suggest that lncRNAs have potential in diagnostic or prognostic. Moreover, they are engaged in the thyroid carcinogenesis as below:

*NEAT1*

Thyroid carcinogenesis can be triggered by NEAT1 (Nuclear-enriched abundant transcript 1), which is shown in various cancers. However, little is known of its involvement in TC. NEAT1 overexpression has been linked to down regulation of miR-214 in TC. Down regulation of miR-214 mediated by NEAT1 results in increased cell motility and survival in in vitro as well as in vivo. NEAT1 overexpression promotes proliferation of TC via modulation of Wnt-beta catenin axis (Zhang et al., 2018b). This recent finding suggests the oncogenic properties of lncRNA NEAT1 in TC biology. Moreover, a recent study has studied the role of NEAT1 in PTC. Sun et al demonstrated that two isoforms of NEAT (NEAT 1 and NEAT2) modulated the expression of ATPase family AAA domain-containing protein 2 (ATAD2) an oncogene via down-regulation of the expression of miR-106-5p. Knockdown of either NEAT1 or NEAT2 in PTC cell lines resulted in growth arrest and inhibition of metastasis. These findings suggest that NEAT1 and NEAT2 both could be used as a diagnostic and therapeutic tool in PTC (Sun et al., 2018). Zhang et al., (2018a) have reported the interplay between NEAT1 and microRNAs. Using bioinformatic approaches, qRT-PCR and Western blot, they determined oncogenic properties of NEAT1. NEAT1 modulated cellular growth and differentiation of PTC cells via upregulation of KLK7. KLK7 is a downstream protein highly expressed in PTC and is regulated by miR-129-5p. NEAT1 overexpression competes with miR-129-5p to inhibit its expression. Consequently, when KLK7 expression is upregulated, it promotes growth and proliferation of PTC in the absence of miR-129-5p. Overexpression of miR-129-5p significantly reduced the tumor via down regulation of NEAT1. These findings suggest that NEAT1 may be a suitable diagnostic as well as therapeutic target in PTC. 

*HOTAIR*

HOTAIR (HOX transcript of antisense RNA) is another important lncRNA that has been reported to be overexpressed in a plethora of cancer types. However, limited data is available on the role of HOTAIR in TC. A recent study has indicated the oncogenic role of HOTAIR in PTC. SNP based analysis conducted by Zhu et al demonstrated that SNP variation in HOTAIR (rs920778 SNP) has been importantly affiliated with down regulation of the Wnt-beta catenin pathway in PTC cell lines. This down regulation resulted in increased cell survival and proliferation indicating that HOTAIR could be implicated in oncogenesis (Zhu et al., 2016). Overexpression of HOTAIR promptly decreased the overall survival in PTC patients via modulation of Wnt signaling cascade (Murugan et al., 2018). Another study conducted by Di et al., (2017) demonstrated that HOTAIR plays a pivotal role in the development and differentiation of PTCs. Using functional assays and nude mice model they showed suppression of HOTAIR with antisense RNAs which resulted in growth arrest. Overexpression of HOTAIR increased the expression of CCND2 a tumor promoting protein under the influence of miR-1. This revealed that HOTAIR could be utilized as a diagnostic approach to understand tumor pathology in PTC. Furthermore, exploring the role miR-1/HOTAIR interaction can open new fields for the development of therapeutic strategies. In a recent microarray-based study on the role of HOTAIR along with ROR and PVT1 in PTC has been evaluated. HOTAIR, PVT1 and ROR modulate protein expression of PTC and their upregulation increases the cell proliferation in PTC (Zhang et al., 2018c). These findings showed that HOTAIR as a useful diagnostic tool for the detection of disease progression in PTC. 

*MALAT1*

MALAT1 (Metastasis-associated lung adenocarcinoma transcript 1) has been investigated extensively in TC. MALAT1 targets IQGAP1. Overexpression of MALAT1 resulted in increased cell growth and invasion in vitro. Huang et al., (2016) showed that MALAT1 expression aid in the induction of tumor growth in mice model, antagonistically acting on IQGAP1, indicating the crucial involvement of MALAT1 in TC. Furthermore, MALAT1 overexpression has been related to EMT transition in TPC1 cell lines under the influence of TGF beta action suggesting a role of MALAT1 in tumor metastasis (Zhang et al., 2017). In situ hybridization indicated that MALAT1 and miR-885 interplay one each other in follicular and Hurthle cell thyroid (HTC) neoplasms. Increased expression of both was observed in FTC as well HTC compared to normal tissue. Furthermore, phosphorylation of mammalian target of Rapamycin (mTOR) was also overexpressed by MALAT1 and miR-885 activity in FTC. These findings suggest that this pathway could be targeted for possible drug delivery system (Covach et al., 2017). Tumor-associated macrophages (TAM), could be elevated in PTC as well as other TC cells, inducing thyroid cells proliferation and angiogenesis. Huang et al demonstrated that MALAT1 directly promoted angiogenesis in TAM by interacting with FGF2. Using CCK8 assay and transwell assay they reported that MALAT1and FGF2 expression was quite high in thyroid tissue as compared to normal control. Moreover, when the expression of MALAT1 and FGF2 was decreased in the FTC133 cells, proliferation, migration and invasiveness were increased explicitly. The interplay between MALAT1 and FGF2 resulted in the decreased inflammation and increased vascularization of TC in vitro (Huang et al., 2017). A qRT-PCR study conducted by Liu et al demonstrated the involvement of MALAT1 in PTC. Expression of MALAT1 confirmed that it played a diverse role in tumor prevalence and carcinogenesis of PTC in vivo. Correlation between tumor size, morphology and other characteristics compared to MALAT1 expression indicated that MALAT1 could be used as a diagnostic tool for the detection of PTC (Liu et al., 2018a).

*ANRIL*

ANRIL (Antisense non-coding RNA in INK4 locus) has been recently established as an oncogenic lncRNA. A study conducted by Zhao et al., (2016) demonstrated that ANRIL was highly overexpressed in TC. ANRIL modulates key signaling cascade such as the TGF beta which in turn inhibits apoptosis by hindering the activity of Smads. Cell line experiments further revealed inhibition of ANRIL in TPC-1 cell lines using siRNA approach which resulted in growth arrest and increased activity of Smads. 

*BANCR*

BANCR (BRAF-activated lncRNA) has been documented to play a crucial role in the differentiation of thyroid tumor cells either by promoting cellular proliferation or growth arrest. BANCR overexpression is a benchmark in TC. BANCR upregulates TSHR which in turn promotes expression of Cyclin D1 that can lead to tumorigenesis (Zheng et al., 2016). A recent study conducted by Liao et al., (2016) demonstrated the double side role of BANCR in PTC cell lines and mice models. There was a reduction in PTC cell growth and increased apoptosis via overexpression of BANCR, also in xenograft mouse models. These results suggested that BANCR could inhibit tumorigenesis in PTC and that BANCR levels could be used as a novel prognostic marker. Moreover, BANCR promotes cellular growth and differentiation in thyroid cells by directly modulating the expression of E-cadherin. This modulation results in the expression of EMT machinery that in turn promotes tumor metastasis and invasion in PTC (Su et al., 2015) . Recently it has been brought to light that BANCR can modulate RAF/MEK/ERK signaling pathway (Wang et al., 2018b). 

*H19*

A study investigated the role of H19 in PTC, whose overexpression leads to the poor prognosis of PTC. Moreover, interplay between estrogen receptor (ER) and estradiol E2 resulted in overexpression of H19 in PTC stem cells. However, H19 silencing decreased ER-beta expression and reduced tumor progression in PTC stem cells indicating the fact that ER-beta and H19 down regulation via E2 could be used as a therapeutic approach to reduce tumor progression in PTC (Chu et al., 2014). In addition, H19 has been shown to interact with insulin receptor substrate 1: this interaction promptly reduced cell growth and migration in various thyroid cell lines. H19 mediated negative down regulation of insulin receptor by inactivation of PI3 kinase/Akt and nuclear factor Kappa B pathway. Owing to these characteristics, H19 may be potentially used as a therapeutic target for TC (Wang et al., 2017). Using qPCR, it was they demonstrated that down-regulation of H19 was responsible of reduced metastatic potential in PTC and thus H19 expression could be utilized as diagnostic marker for PTC (Jiao et al., 2019).

*XLOC_051122 and XLOC_006074*

XLOC_051122 and XLOC_006074 are two recently identified lncRNAs that have been reported to be overexpressed PTC in correlation to BRAF mutations. Overexpression of either XLOC_051122 or XLOC_006074 greatly increased TC widespread to lymph nodes irrespective of BRAF mutations in PTC (Liyanarachchi et al., 2016). 

*HIT000218960, LOC100507661, ENST00000537266 and ENST00000426615*

Other lncRNAs, the HIT000218960, LOC100507661, ENST00000537266 and ENST00000426615, have also been investigated in TC. They showed to play a role in tumor biology, even if only preliminary results are available (Kim et al., 2016; Xu et al., 2016; Li et al., 2017). Targeting these lncRNAs may have promising benefits for tumor eradication. 

*Potential applications of lncRNAs in TC*

A number of lncRNAs have been reported to be overexpressed in tissue and cancer specific manner. Certain lncRNAs show high expression in a specific tissue while others have fairly low expression in malignant tissue. Such apparently contrasting expression entails lncRNAs as an excellent diagnostic marker for evaluating disease progression. Oncogenic lncRNAs overexpression is a clear signature for several tumors. The susceptibility of lncRNAs as a diagnostic marker for various human pathologies has been well established given their existence in body fluids. 

For example, in human cancer, lncRNAs have been showed to be useful potential diagnostic markers: lncRNA XLOC_009167 was screened as a candidate biomarker for lung cancer by Jiang et al., (2018). Their findings showed that lncRNA XLOC_009167 was hugely overexpressed in human blood and gave a better idea of disease progression compared to other current available diagnostic tools suggesting that lncRNAs could be utilized as an essential tool for detection as well as monitoring lung cancer (Jiang et al., 2018). In hepatocellular carcinoma (HCC), another oncogenic lncRNA, uc007biz.1 (LRB1) has been reported to act as a marker for the determining disease pathogenicity (Wang et al., 2018d). Elevated serum levels of LRB1 was found to be associated with the disease progression in the 326 patients with HCC compared to 73 control patients. Furthermore, LRB1 expression was tightly linked with alpha-fetoprotein. A combination of LRB1, AFP and des-y-carboxyprothrombin (DCP) could be used as a diagnostic marker for the detection of cell damage in HCC (Wang et al., 2018c). DUXAP8 and LINC00460 are the two lncRNAs that can have a potential diagnostic role for the detection of esophageal cancer (Liu et al., 2018c). Furthermore, PCA3 has been overexpressed in prostate cancer. This overexpression compared to normal cells has proven to be an excellent diagnostic marker in prostate tumors (Lee et al., 2011). LncRNA HOTTIP (HOXA distal transcript antisense RNA) an active oncogenic lncRNA whose expression is strictly related to the metabotropic glutamate receptor 1 (mGluR1) has been demonstrated to be used as an effective diagnostic marker for pancreatic cancer (Wang et al., 2018a). In addition lncRNA, UCA1 has been involved in bladder cancer, while HULC in liver pathologies and AA174084 in oral cancers (Murugan et al., 2017).

Indeed CCND2-AS1 lncRNA is highly expressed in PTC and gain or loss of function mutation of CCND2-AS1 resulted in deregulated cellular growth, migration and invasion of PTC. Owing to these peculiarities CCND2-AS1 can be used as diagnostic as well as a prognostic marker for the PTC (Xia et al., 2018). LncRNAs cancer susceptibility candidate 2 (CASC2) has been demonstrated to be correlated to tumorigenesis of a large number of tumors. Overexpression of CASC2 has been showed to trigger growth arrest in TC indicating the fact that CASC2 could be used as diagnostic as well as a prognostic marker (Xiong et al., 2017). NONSHAT037832 down regulation is indispensable for understanding tumor physiology and TNM staging of PTC. Lower levels of expression of NONSHAT037832 in PTC cell line such as the IHH-4 suggested its importance as a prognostic marker (Murugan et al., 2018). rs2439302 is another lncRNA whose down-regulation has been affiliated with a greater survival rate in PTC patients (Murugan et al., 2018). In addition, lncRNA-NR_036575.1 overexpression in extrathyroidal extension (ETE) and tumor size maintenance has managed it as an essential prognostic marker for both PTC as well as ATC (Sun et al., 2016). XLOC_051122 and XLOC_006074, two lncRNAs have been reported to be overexpressed in PTC and other TC such as the FTC and MTC. Recent evidence showed the correlation between these lncRNAs and BRAF mutations. XLOC_051122 and XLOC_006074 positively regulate BRAF axis resulting in cell growth and differentiation of tumor cells. This lead to adverse prognosis and dis-oriented TNM grading (Liyanarachchi et al., 2016). Lately SLC6A9-5:2 expression has been related to iodine resistant TC. Lower expression of SLC6A9-5:2 increased the levels of PARP-1 that could result in triggering tumor growth and proliferation with a consequently poor prognosis. Owing to these characteristics SLC6A9-5:2 could be used as prognosis biomarker in iodine resistant TC (Xiang et al., 2017). Altogether these findings stress on the use of lncRNAs for development of diagnostic and prognostic tools in TC. 

In conclusion, lncRNAs can be considered the regulator of TC. Aberrant expression of lncRNAs in TC may lead to altered physiology and have provided significant clues on the mechanism of tumorigenesis. 

**Figure 2:**
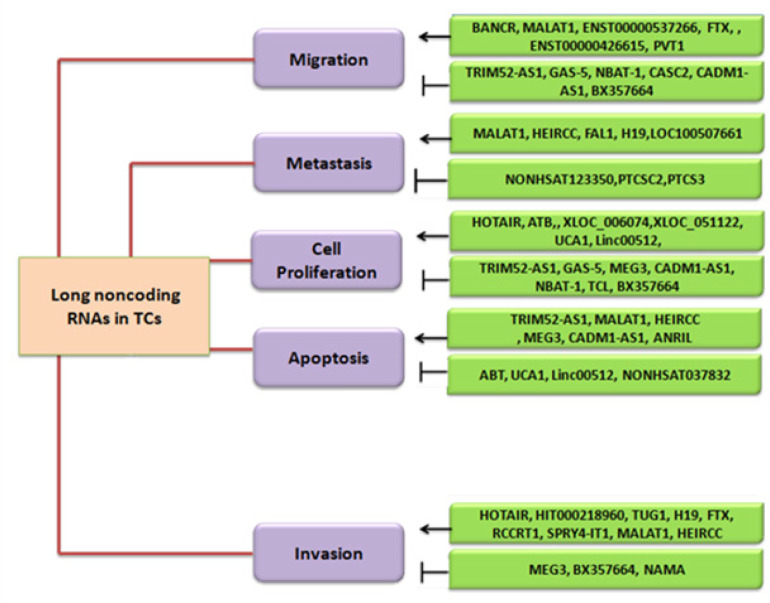
Functions of lncRNAs in the Pathogenesis and Their Potential Clinical Applications or Therapeutic Strategies in TC. LncRNAs play a vital role in Cell Migration, Metastasis, Cell Proliferation, Apoptosis, Cell cycle process and Invasion. Arrows indicate the inhibition and activation of these mechanisms by lncRNAs and how these lncRNAs can be exploited for the therapeutic effects

## Conflict of Interest

There is no conflict of interest. 

## References

[B1] Aboelnaga EM, Ahmed RA (2015). Difference between papillary and follicular thyroid carcinoma outcomes: an experience from Egyptian institution. Cancer Biol Med.

[B2] Alok Pathak K, Leslie WD, Klonisch TC (2013). The changing face of thyroid cancer in a population-based cohort. Cancer Med.

[B3] Bhan A, Mosella M, Singer MC (2018). DNA methylation signature associated with risk of recurrence in papillary thyroid carcinoma. Thyroid.

[B5] Chu R, Van Hasselt A, Vlantis AC (2014). The cross-talk between estrogen receptor and peroxisome proliferator-activated receptor gamma in thyroid cancer. Cancer.

[B6] Covach A, Patel S, Hardin H (2017). Phosphorylated Mechanistic target of Rapamycin (p-mTOR) and Noncoding RNA expression in follicular and Hürthle cell thyroid neoplasm. Endocr Pathol.

[B7] Derrien T, Johnson R, Bussotti G (2012). The GENCODE v7 catalog of human long noncoding RNAs: analysis of their gene structure, evolution, and expression. Genome Res.

[B8] Di W, Li Q, Shen W (2017). The long non-coding RNA HOTAIR promotes thyroid cancer cell growth, invasion and migration through the miR-1-CCND2 axis. Am J Cancer Res.

[B9] Guerra G, Cinelli M, Mesolella M (2014). Morphological, diagnostic and surgical features of ectopic thyroid gland: a review of literature. Int J Surg.

[B10] Gutschner T, Diederichs S (2012). The hallmarks of cancer: a long non-coding RNA point of view. RNA Biol.

[B11] Guttman M, Amit I, Garber M (2009). Chromatin signature reveals over a thousand highly conserved large non-coding RNAs in mammals. Nature.

[B12] Hangauer MJ, Vaughn IW, McManus MT (2013). Pervasive transcription of the human genome produces thousands of previously unidentified long intergenic noncoding RNAs. PLoS Genet.

[B13] Huang J-k, Ma L, Song W-h (2016). MALAT1 promotes the proliferation and invasion of thyroid cancer cells via regulating the expression of IQGAP1. Biomed Pharmacother.

[B14] Huang Jk, Ma L, Song Wh (2017). LncRNA-MALAT1 promotes angiogenesis of thyroid cancer by modulating tumor-associated macrophage FGF2 protein secretion. J Cell Biochem.

[B15] Jalali S, Bhartiya D, Lalwani MK (2013). Systematic transcriptome wide analysis of lncRNA-miRNA interactions. PLoS One.

[B16] Jiang N, Meng X, Mi H (2018). Circulating lncRNA XLOC_009167 serves as a diagnostic biomarker to predict lung cancer. Clin Chim Acta.

[B17] Jiao X, Lu J, Huang Y (2019). Long non-coding RNA H19 may be a marker for prediction of prognosis in the follow-up of patients with papillary thyroid cancer. Cancer Biomarkers.

[B18] Joo LJS, Zhao JT, Gild ML (2018). Epigenetic regulation of RET receptor tyrosine kinase and non-coding RNAs in MTC. Mol Cell Endocrinol.

[B19] Kilfoy BA, Zheng T, Holford TR (2009). International patterns and trends in thyroid cancer incidence, 1973–2002. Cancer Causes Control.

[B20] Kim D, Lee WK, Jeong S (2016). Upregulation of long noncoding RNA LOC100507661 promotes tumor aggressiveness in thyroid cancer. Mol Cell Endocrinol.

[B21] Lamartina L, Grani G, Durante C (2018). Follow-up of differentiated thyroid cancer–what should (and what should not) be done. Nat Rev Endocrinol.

[B22] Lan X, Zhang H, Wang Z (2015). Genome-wide analysis of long noncoding RNA expression profile in papillary thyroid carcinoma. Gene.

[B23] Lee GL, Dobi A, Srivastava S (2011). Prostate cancer: diagnostic performance of the PCA3 urine test. Nat Rev Urol.

[B24] Li T, Yang X-d, Ye C-x (2017). Long noncoding RNA HIT000218960 promotes papillary thyroid cancer oncogenesis and tumor progression by upregulating the expression of high mobility group AT-hook 2 (HMGA2) gene. Cell Cycle.

[B25] Liao T, Qu N, Shi R-L (2017). BRAF-activated LncRNA functions as a tumor suppressor in papillary thyroid cancer. Oncotarget.

[B26] Liu J, Dong H, Yang Y (2018a). Upregulation of long noncoding RNA MALAT1 in papillary thyroid cancer and its diagnostic value. Future Oncol.

[B27] Liu L, Wu B, Cai H (2018b). Tiam1 promotes thyroid carcinoma metastasis by modulating EMT via Wnt/β-catenin signaling. Exp Cell Res.

[B28] Liu W, Zhang Y, Chen M (2018c). A genome-wide analysis of long noncoding RNA profile identifies differentially expressed lnc RNA s associated with Esophageal cancer. Cancer Med.

[B29] Liyanarachchi S, Li W, Yan P (2016). Genome-wide expression screening discloses long noncoding RNAs involved in thyroid carcinogenesis. J Clin Endocrinol Metab.

[B30] Murugan AK, Munirajan AK, Alzahrani AS (2017). Long noncoding RNAs: emerging players in thyroid cancer pathogenesis. Endocr Relat Cancer.

[B31] Murugan AK, Munirajan AK, Alzahrani AS (2018). Long noncoding RNAs: emerging players in thyroid cancer pathogenesis. Endocr Relat Cancer.

[B32] Orosco RK, Hussain T, Noel JE (2019). Radioactive iodine in differentiated thyroid cancer: a national database perspective. Endocr Relat Cancer.

[B33] Pellegriti G, Frasca F, Regalbuto C (2013). Worldwide increasing incidence of thyroid cancer: update on epidemiology and risk factors. J Cancer Epidemiol.

[B34] Schwarzenbach H, Hoon DS, Pantel K (2011). Cell-free nucleic acids as biomarkers in cancer patients. Nat Rev Cancer.

[B35] Solomon TN, Oli PT, Kailas CK (2019). Prevalence of thyroid malignancy in goitre: a cross sectional study. Int Surg J.

[B36] Starren J, Williams MS, Bottinger EP (2013). Crossing the omic chasm: a time for omic ancillary systems. JAMA.

[B37] Su S, Gao J, Wang T (2015). Long non-coding RNA BANCR regulates growth and metastasis and is associated with poor prognosis in retinoblastoma. Tumor Biol.

[B38] Sun W, Lan X, Wang Z (2016). Overexpression of long non-coding RNA NR_036575 1 contributes to the proliferation and migration of papillary thyroid cancer. Med Oncol.

[B39] Sun W, Lan X, Zhang H (2018). NEAT1_2 functions as a competing endogenous RNA to regulate ATAD2 expression by sponging microRNA-106b-5p in papillary thyroid cancer. Cell Death Dis.

[B40] Trimboli P, Ulisse S, Graziano F (2006). Trend in thyroid carcinoma size, age at diagnosis, and histology in a retrospective study of 500 cases diagnosed over 20 years. Thyroid.

[B41] Wang P, Liu G, Xu W (2017). Long noncoding RNA H19 inhibits cell viability, migration, and invasion via downregulation of IRS-1 in thyroid cancer cells. Technol Cancer Res Treat.

[B42] Wang X, Xiao L, Yu H (2018a). Expression levels of long noncoding RNA HOXA distal transcript antisense RNA and metabotropic glutamate receptor 1 in pancreatic carcinoma, and their prognostic values. Oncol Lett.

[B43] Wang Y, Gu J, Lin X (2018b). lncRNA BANCR promotes EMT in PTC via the Raf/MEK/ERK signaling pathway. Oncol Lett.

[B44] Wang ZF, Hu R, Pang JM (2018c). Serum long noncoding RNA LRB1 as a potential biomarker for predicting the diagnosis and prognosis of human hepatocellular carcinoma. Oncol Lett.

[B46] Xia E, Bhandari A, Shen Y (2018). LncRNA CCND2-AS1 promotes proliferation, migration, and invasion in papillary thyroid carcinoma. Biochem Biophys Res Commun.

[B47] Xiang C, Zhang M-L, Zhao Q-Z (2017). LncRNA-SLC6A9-5:2: A potent sensitizer in 131I-resistant papillary thyroid carcinoma with PARP-1 induction. Oncotarget.

[B48] Xiong X, Zhu H, Chen X (2017). Low expression of long noncoding RNA CASC2 indicates a poor prognosis and promotes tumorigenesis in thyroid carcinoma. Biomed Pharmacother.

[B49] Xu B, Shao Q, Xie K (2016). The long non-coding RNA ENST00000537266 and ENST00000426615 influence papillary thyroid cancer cell proliferation and motility. Cell Physiol Biochem.

[B50] Zhang H, Cai Y, Zheng L (2018a). Long noncoding RNA NEAT1 regulate papillary thyroid cancer progression by modulating miR-129-5p/KLK7 expression. J Cell Physiol.

[B51] Zhang M, Weng W, Zhang Q (2018b). The lncRNA NEAT1 activates Wnt/β-catenin signaling and promotes colorectal cancer progression via interacting with DDX5. J Hematol Oncol.

[B52] Zhang R, Hardin H, Huang W (2018c). Long non-coding RNA Linc-ROR is upregulated in papillary thyroid carcinoma. Endocr Pathol.

[B53] Zhang R, Hardin H, Huang W (2017). MALAT1 long non-coding RNA expression in thyroid tissues: analysis by in situ hybridization and real-time PCR. Endocr Pathol.

[B54] Zhao J-J, Hao S, Wang L-L (2016). Long non-coding RNA ANRIL promotes the invasion and metastasis of thyroid cancer cells through TGF-β/Smad signaling pathway. Oncotarget.

[B55] Zheng H, Wang M, Jiang L (2016). BRAF-activated long noncoding RNA modulates papillary thyroid carcinoma cell proliferation through regulating thyroid stimulating hormone receptor. Cancer Res Treat.

[B56] Zhu H, Lv Z, An C (2016). Onco-lncRNA HOTAIR and its functional genetic variants in papillary thyroid carcinoma. Sci Rep.

